# Kinetic Improvement of Bioactive Compounds Extraction from Red Grape (*Vitis vinifera* Moldova) Pomace by Ultrasonic Treatment

**DOI:** 10.3390/foods8080353

**Published:** 2019-08-17

**Authors:** Florina Dranca, Mircea Oroian

**Affiliations:** Faculty of Food Engineering, Stefan cel Mare University of Suceava, 720229 Suceava, Romania

**Keywords:** grape pomace, extraction, total monomeric anthocyanin, total phenolic content

## Abstract

Grape pomace from a red grape variety (*Vitis vinifera* Moldova) cultivated in the northeastern region of Romania has been studied as a source for the extraction of total monomeric anthocyanin (TMA) and total phenolic content (TPC) using ultrasonic treatment. The method of extraction described here uses two different solvents, namely 2-propanol and methanol. For each of the extraction solvents, we evaluated the singular influence and the impact of interactions between process parameters (solvent concentration, ultrasonic frequency, temperature, and extraction time) on the extraction yields of anthocyanins and phenolic compounds. Response surface methodology was implemented via a Box–Behnken design to optimize the extraction of TMA and TPC from grape pomace. According to the optimization, in order to achieve the highest yield of TPC (62.487 mg gallic acid equivalent (GAE)/g (d = 1.0)), the following conditions are necessary: solvent—2 propanol, solvent concentration 50%, temperature −50 °C and extraction time 29.6 min.

## 1. Introduction

Grapes (genus *Vitis*) are one of the most widely cultivated fruit crops in the world, with uses in the food industry that range from juice, wine, jam, jelly, raisins, and vinegar production to oil extraction from grape seeds. It is estimated that about 80% of the grape harvest is used in the winemaking industry [1]. Wine production and consumption has traditionally been concentrated in the European continent, and continues to be dominated by three major European producers: Italy, France and Spain. Since its major decline in 2012 [2], global wine production rose to 275.7 MhL in 2015, with a slight increase of 2% compared with the previous year [3]. As can be expected, such a large industry is associated with the production of significant quantities of solid and liquid waste. Solid waste includes grape stalks (2.5–7.5%), grape pomace (up to 25–45%) and grape seeds (3–6%) [4]. In European countries alone, it has been estimated that 14.5 million tons of grape by-products are generated annually by winemaking industry [5,6].

Grape pomace is the main winery waste, and results from the winemaking process during the production of grape must by crushing whole grapes. Grape pomace, which is comprised of skins and seeds, is reported to account for an approximate percentage of between 25% and 30% of the grapes used [7,8]. The reported content of seeds in grape pomace varies from 7–20% to a greater proportion of 38–52% [9,10]. Regarding the chemical composition of grape pomace, several studies reported on the content in protein, fiber, oil, and sugars of the by-product [11,12,13,14]. Due to its high fiber content, grape pomace is being used as a feed additive [15]. Other reuses described for this winery waste are as raw materials in the production of alcoholic beverages and fertilizers, and also in distilleries for obtaining ethanol [16,17,18].

Grapes contain nutrient elements (carbohydrates, edible fibers, vitamins, minerals, etc.) and also phytochemicals, the most important being phenolic compounds. From this class of compounds, predominant in grapes are anthocyanins, flavanols, flavonols, and resveratrol [19]. Anthocyanins are the main flavonoids accumulated in grape cultivars with colored skins [20,21]. According to concentration, the most abundant anthocyanins found in grape skin are malvidin-3-*O*-glucoside and peonidin-3-*O*-glucoside [22,23]. As one of the most ubiquitous classes of flavonoids in red grapes, anthocyanins also play significant roles in the color of their main product, red wine [24]. As mentioned before, grape must is obtained by crushing and pressing whole grapes. These mechanical processes lead to an incomplete extraction of grape phenolics, and therefore a significant amount of phenolics passes into must, while certain amounts remain in the pomace. Previous research has shown that phenolic compounds remain in grape pomace in a proportion of about 70% [25,26]. The most abundant phenolic classes determined in grape pomace extracts were anthocyanins, followed by flavan-3-ols, phenolic acids (predominantly hydroxycinnamic acids) and flavonols [27]. Phenolic compounds have potential beneficial effects on human health because of their antioxidant activity and antimicrobial, antiviral, and anti-inflammatory properties [28]. From this point of view, the extraction of these bioactive substances can provide many opportunities for adding value to food products, and thereupon contribute to the improvement of dietary pattern of the population [29].

Extraction is an important step in the recovery of phenolic compounds from plant materials prior to their use. There is no standard extraction method that has been developed for this particular purpose, and as a consequence, several extraction techniques have been applied. The widespread technique used to extract phenolic compounds from grape pomace is solid–liquid extraction; industrial scale solid–liquid extraction combines water with other solvents such as ethanol, methanol or sulphur dioxide [30]. To achieve a reduction in the extraction time and the consumption of organic solvents, both while maintaining a high recovery of phenolic compounds, novel techniques of extraction have been proposed and successfully applied for winery waste capitalization. The non-conventional extraction techniques with applications documented for grape pomace are: supercritical fluids extraction (SFE) [31,32,33], microwave assisted extraction (MAE) [34,35,36], high-pressure and -temperature extraction (HPTE) [37,38], and ultrasound-assisted extraction (UAE) [39,40]. Microwave- and ultrasound-assisted extractions, particularly, are recognized as outstanding techniques that enhance the extraction of phenolic compounds. Advantages of ultrasonic treatment include higher productivity, yield and selectivity, better processing time, enhanced quality, reduced chemical and physical hazards, and the fact that this technique is environmentally friendly [41]. Moreover, UAE appears to be much faster than traditional procedures, because it provides a higher contact-surface area between solid and liquid phase as a consequence of the fine reduction of particle size [42].

In the present work, we study the influence of different extraction conditions, namely extraction temperature, ultrasonic frequency, solvent concentration, and sonication time, on the content in anthocyanins and phenolic compounds of extracts from grape pomace resulting from processing red grapes of a local variety (*Vitis vinifera* Moldova)at lab scale. The changes induced in the extract composition as an effect of variations in operating variables were quantified by measuring TMA and TPC of extracts and Raman spectroscopy was used for the characterization of the extracts.

## 2. Materials and Methods

### 2.1. Materials and Chemicals

Red grapes (*Vitis vinifera* Moldova) were purchased in Suceava city (Romania) in 2015. The grape pomace (after the extraction of the juice) was submitted to a drying process at 40 °C in an oven until it was a constant weight. The dried grape pomace was then milled until a fine powder was obtained.

All chemical reagents—methanol, 2-propanol, sodium carbonate and Folin–Ciocalteau reagent—were of analytical grade and were purchased from Sigma-Aldrich (Hamburg, Germany).

### 2.2. Extraction Technique

3 g of dried grape pomace powder were mixed with 30 mL of solvent (methanol or 2-propanol in different concentrations with water 50% (*v*/*v*), 70% (*v*/*v*), and 90% (*v*/*v*), respectively). The ultrasonic procedure was a direct one using an ultrasonic transducer coupled with a function generator (HAMEG 8150, Munich, Germany) and the frequency was swept at three different frequencies (Table 1). The samples were extracted at three different temperatures (50, 60 and 70 °C) for 15 min, 30 min and 45 min, respectively.

### 2.3. Total Monomeric Anthocyanin Determination

The total monomeric anthocyanin (TMA) content of grape pomace extract was determined using a spectrophotometric method developed by Rabino and Mancinelli [43]. A UV-VIS spectrophotometer (Ocean Optics, Largo, FL, USA) was used for spectral measurements at 657 nm and 530 nm. 

TMA content was expressed in cyanindin-3-glucoside (mg/g) using the following equation:

(1) $$Anthocyanin\left(\frac{mg}{g}\right)=\frac{A_{net}}{29600}\cdot MW\cdot DF\cdot \frac{V}{Wt}$$

where: *MW*= 449.1, *A_net_*= *A_530_*− 0.25 *A_657_*, *DF*-dilution factor, *V*-total volume (mL), *Wt*-sample weight (g).

### 2.4. Total Phenolic Content

The total phenolic content of crude extracts was measured using the Folin–Ciocalteu (FC) reagent-based colorimetric assay as described by Singleton and Rossi [44]. The TPC values were expressed in mg gallic acid equivalent (GAE)/g [45] in extract wet basis. The calibration curve was done using gallic acid at concentrations of 0.5, 1, 2, 3, 4, 5 and 10 mg/L with a regression coefficient of 0.995.

### 2.5. Raman Spectra Analysis

Spectra of grape pomace extracts were recorded using an i-Raman spectrometer (EZM-A2-785L, B&W TEK Inc., Newark, DE, USA) equipped with a fiber-optic Raman probe; the spectrum was recorded from 2600 − 175 cm^−1^. Samples were scanned at an increment of 10 nm. Integration time was 15 s. In order to display the spectra of the extract, Spekwin32-optical spectroscopy software (Version 1.72.2, 2016, http://www.effemm2.de/spekwin/) was used.

### 2.6. Statistical Analysis

#### 2.6.1. Analysis of Variance

Multifactor analysis of variance (ANOVA) was applied to detect differences in the extraction yield of total phenolic content and total monomeric anthocyanins with changes in the process parameters, namely, extraction solvent, solvent concentration, ultrasonic frequency, temperature, and extraction time. Analyses were performed with the Unscrambler X 10.1 (Camo, Oslo, Norway). Differences were considered to be significant at *p* < 0.05.

#### 2.6.2. Response Surface Methodology (RSM)

A 3^4^ full factorial experiment was conducted for the solvents used (2-propanol and methanol) with 4 factors (ultrasonic frequency, temperature, solvent concentration, and time), each varying at 3 levels, as presented in Table 1. The input variables of the design were TMA and TPC. The design was performed using Design Expert 10.0.3.1 (trial version, Minneapolis, MN, USA).

It was a Box-Behnken design based on a second-order (quadratic) polynomial response surface model using the following equation:

(2) $$y=b_{0}+\sum_{i=1}^{n}\left(b_{i}x_{i}\right)+\sum_{i=1}^{n}\left(b_{ii}x_{ii}^{2}\right)+\sum_{ij=1}^{n}\left(b_{ij}x_{i}x_{j}\right)$$

where: *y* is the predicted response (TMA or TPC), *x_i_* stands for the coded levels of the design variable (solvent concentration, time, temperature and ultrasonic frequency; Table 1), *b*_0_ is a constant, *b_i_* represents the linear effects, *b_ii_* represents the quadratic effects and *b_ij_* represents the interaction effects.

The parameters were optimized in order to achieve the highest concentration of TMA and TPC using the desirability function approach [46].

## 3. Results and Discussion

### 3.1. Influence of Extraction Parameters on TMA and TPC Yield

The targeted compounds were subsequently extracted by ultrasonication with two different solvents, namely, 2-propanol and methanol. The influence of extraction solvent selection on the extraction yield was further studied by varying solvent concentration and recording the changes in the solubility of phenolics and anthocyanins. Similarly, the effect of increasing extraction temperature and extending the sonication time was also investigated. The influence of the parameters of TMA and TPC are presented in Table 2, and the interactions between the extraction parameters are presented in Table 3. 

#### 3.1.1. Solvent Type and Concentration

Solvent type is one of the main factors affecting the extraction efficiency of phenolic compounds from plant materials. Due to the polar nature of phenolic compounds, the extraction is usually performed in polar protic media, in which these compounds are easily solubilized. Two polar solvents, namely 2-propanol and methanol, were used in this study, and their ability to extract anthocyanins and phenolic compounds was compared. As Table 2 shows, 2-propanol gave higher extraction yields of total monomeric anthocyanin (average concentration of 5.37 mg/g grape pomace) in comparison to methanol (average of 4.58 mg/g). As a result, the choice of extraction solvent had a significant effect on the extraction yield of TMA (*p* < 0.001). Comparable extraction yields of anthocyanins were obtained by Corrales et al. [37]. Applying an ultrasonic treatment to grape pomace dispersed in a mixture of ethanol and water (50:50) at a frequency of 35 kHz at 70 °C resulted in extraction yields of 7.76 mg/g for total anthocyanins and 4.85 mg/g for total anthocyanin monoglucosides. Through the conventional extraction of dehydrated grape pomace from the red grape variety Benitaka (São João do Piauí, Piui, Northeastern Brazil) in a solution of ethanol 85% and HCl (1.5 N) for 24 h under refrigeration and absence of light, Sousa et al. [29] determined the content of anthocyanins to be 131 mg/100 g. Compared to the content of anthocyanins measured in the current study, the levels reported by the above-mentioned authors are approximately 4 times lower; the differences are justified by the variation in anthocyanin content between varieties [47] and the choice of extraction solvent, and can be also attributed to a degradation of these compounds due to the high temperature (60 °C) applied in the drying process of pomace, as it was previously indicated that high temperature (maximum 35 °C) causes a reduction in total anthocyanin content [48].

In the case of total phenolic content, both extraction solvents ensured an efficient extraction, with methanol being particularly remarkable for its high extraction yield (average of 46.53 mg/g). Similar to total monomeric anthocyanin extraction, the solvent type had a significant effect on the extraction yield (*p* < 0.001). The highest levels of phenolic compounds using methanol as an extraction solvent were also obtained by Casazza et al. [49], who determined a total content of phenolics of 90 mg/g in dried Pinot Noir skins when using UAE. The affinity of phenolic compounds for methanol may be ascribed to the dielectric constant (32.60), which is greater than that of 2-propanol (19.90) [50], or to the lower surface tension of methanol (22.1 mJ/cm^2^) when compared to 2-propanol (23.3 mJ/cm2) [51]. For the same red grape variety (*Vitis vinifera* Moldova) Budiul and Albulescu [52] reported a total phenol content of 1164.3 mg/L for Soxhlet extraction with methanol and of 1562.75 mg/L for extraction with a mixture of MeOH:H_2_O:HCl (90:9.5:0.5, *v*/*v*). Drosou et al. [53] previously showed that ultrasonic-assisted extraction of grape pomace leads to greater extraction yields of phenolic compounds than Soxhlet extraction in ethanol or water. Therefore, a great increase in the extraction yield should be expected when using ultrasonic treatment. 

The increase in solvent concentration above 50% caused a decrease in both total monomeric anthocyanin and total phenolic content extraction. The decrease in the extraction yield was particularly pronounced in the case of TMA. Also, solvent concentration had a significant effect on the extraction yield of both TPC and TMA (*p* < 0.001). Several studies describe the use of solvent/water mixtures for the extraction of grape by-products; the addition of water increases the permeability of cell tissue, enabling a better mass transfer by molecular diffusion, as well as the recovery of water-soluble compounds [28,54]. Benmeziane et al. [55] studied the extraction of phenolic compounds from fresh table grapes with distilled water, acetone, methanol, and ethanol. For acetone, methanol and ethanol, the concentration was varied as follows: 20%, 40%, 60%, and 80%. The authors noted an increase in total phenol extraction with the increase in solvent concentration. As in the current study the effect of solvent concentration was opposite, the global effect of other extraction parameters should be considered. In regard to the interactions between solvent concentration and other process parameters, a significant effect of the interactions between solvent concentration and ultrasonic frequency, and concentration and temperature was highlighted (Table 3).

#### 3.1.2. Ultrasonic Frequency

In addition to extraction parameters such as solvent, solvent concentration, solid/liquid ratio, extraction temperature and time, which are common to a wide range of extraction techniques, and have therefore been thoroughly studied, in the case of ultrasound assisted extraction the effect of parameters related to ultrasound wave distribution should be considered. In other words, parameters intrinsically related to the ultrasonic devices (such as the frequency, wavelength and amplitude of the wave), the ultrasonic power (in kWh/L), and consequently, intensity, also have an effect on the extraction [56], and their influence on extraction yield needs to be evaluated. In this study, we recorded the variation in the composition of extracts in phenolic compounds and anthocyanins caused by the change of ultrasonic frequency from 12.5 kHz to 25 KHz, and finally to 37.5 kHz. As can be observed in Table 2, regardless of the compounds targeted by the extraction, an increase of ultrasonic frequency from 12.5 kHz to 25 kHz led to a better solubilization of bioactive compounds in the solvent. In the case of total monomeric anthocyanin, the extraction yield increased by approximately 18% when increasing ultrasonic frequency from 12.5 kHz to 25 kHz. Similarly, the total phenolic content of the extract treated with ultrasounds at a frequency of 25 kHz increased by approximately 11%. A further increase of ultrasonic frequency from 25 kHz to 37.5 kHz resulted in a significant reduction of TMA and TPC content. Consequently, to reach the maximum extraction yield, 25 kHz was the most suitable frequency. Other studies on the extraction of phenolic compounds from grape have reported the application of ultrasonic frequencies of 24 kHz on samples obtained from whole berries and 40 kHz on samples of powdered grape peel [57,58].

#### 3.1.3. Temperature and Time

Extraction temperature and time are important parameters to be optimized in order to achieve high recoveries of anthocyanins and total phenolic compounds, and also to minimize the energy cost of the extraction process. The extraction of TMA and TPC was carried out at 50, 60 and 70 °C. According to the data presented in Table 2, the increase in working temperature from 50 °C to 60 °C had a positive effect on the extraction of anthocyanins from grape pomace. The average concentration of total monomeric anthocyanins measured in the extracts at 60 °C was 5.17 mg/g. However, the positive effects of high temperatures on TMA extraction did not extend to temperatures above 60 °C, as the stability of these compounds decreased leading to lower extraction yields. This reduction of anthocyanin recovery is in agreement with findings of previous studies that demonstrated negative effects of high temperatures (>60 °C) on anthocyanin stability [59,60]. While the change in temperature had both positive and negative effects on TMA, in the case of TPC a continuous negative influence of the increase in this process parameter was observed. For the ultrasound assisted extraction of natural antioxidants from *Jatropha integerrima*, Xu et al. [61] also reported a negative influence of increasing temperature from 40 °C to 80 °C. The reduction in TPC of extracts could be attributed to the degradation of thermolabile and easily oxidizable phenolics caused by simultaneous effects of high temperatures and increased pressures generated when the cavitation bubbles induced by ultrasound reach a critical size [62].

As mentioned before, time is another critical factor in the extraction of phenolic compounds. In this study, the extraction time was gradually increased as follows: 15 min, 30 min and 45 min. In this way, the extraction period needed for achieving maximum yields of total monomeric anthocyanins and total phenolic compounds from grape pomace was about 30 min. The increase in the extraction of targeted compounds at prolonged sonication times has been noted by numerous researchers, and was attributed to the fact that ultrasonic waves affect the mass transfer rate mainly in the solvent penetration stage [63,64]. González-Centeno et al. [65] studied the aqueous ultrasound assisted extraction of antioxidants from grape pomace to investigate the effect of acoustic frequency, ultrasonic power density and extraction time. Regarding the extraction time, researchers reported that the optimal conditions implied a sonication of 25 min. Ghafoor et al. [66] optimized the process variables of UAE of anthocyanins, phenolic compounds and antioxidants from grape seeds, obtaining for the extraction time levels around 30 min, as follows: 29.03 min for phenolic compounds, 30.58 min for antioxidants, and 29.49 for anthocyanins.

Apart from studying the influence of process parameters on extraction yields, statistical analysis was also used for the evaluation of the interactions between factors (Table 3) on extraction yields of TPC and TMA. As shown in Table 3, in the case of TPC, the interactions of solvent type with ultrasonic frequency and of ultrasonic frequency with solvent concentration, and temperature, respectively, had a great impact on extraction efficiency. The impact of these interactions on the extraction yield of phenolic compounds was indicated by the F-values and *p*-values given by the analysis of variance, as follows: solvent type with ultrasonic frequency—F-value of 27.74, *p* < 0.001; solvent concentration with ultrasonic frequency—F-value of 12.80, *p* < 0.001; and ultrasonic frequency with temperature—F-value of 5.82, *p* < 0.001. For TMA extraction, the yield was significantly influenced by the interactions of solvent type with ultrasonic frequency and temperature, respectively, and the interaction between solvent concentration and temperature. Multifactor ANOVA gave the following results for these interactions: solvent type with ultrasonic frequency—F-value of 71.76, *p* < 0.001; solvent type with temperature—F-value of 21.54, *p* < 0.001; and solvent concentration with temperature—F-value of 6.58, *p* < 0.001.

### 3.2. Extraction Modeling

The response surface methodology was implemented via a three-block Box–Behnken experimental design (Table 4) in order to optimize the ultrasonic-assisted extraction of TMA and TPC from grape pomace.

#### 3.2.1. Total Monomeric Anthocyanin

**Figure 1:**
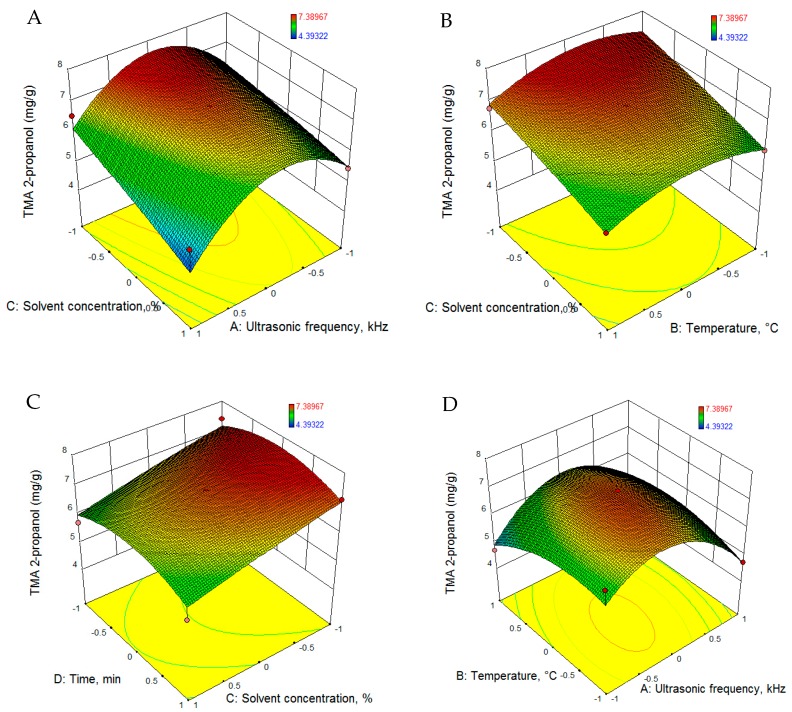

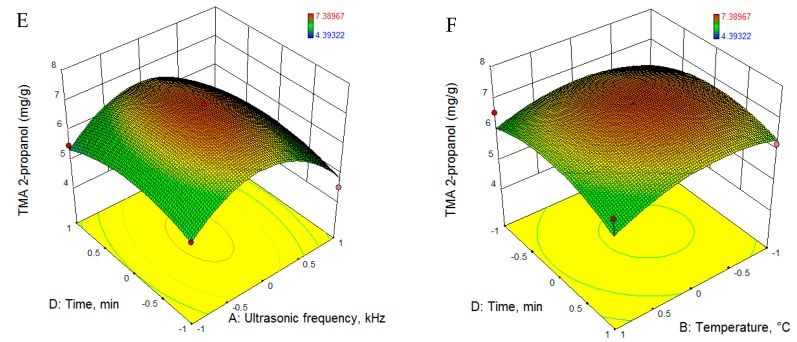
RSM graphs of TMA extraction evolution using 2-propanol as solvent. RSM: Response Surface Methodology, TMA: total monomeric anthocyanin.

**Figure 2:**
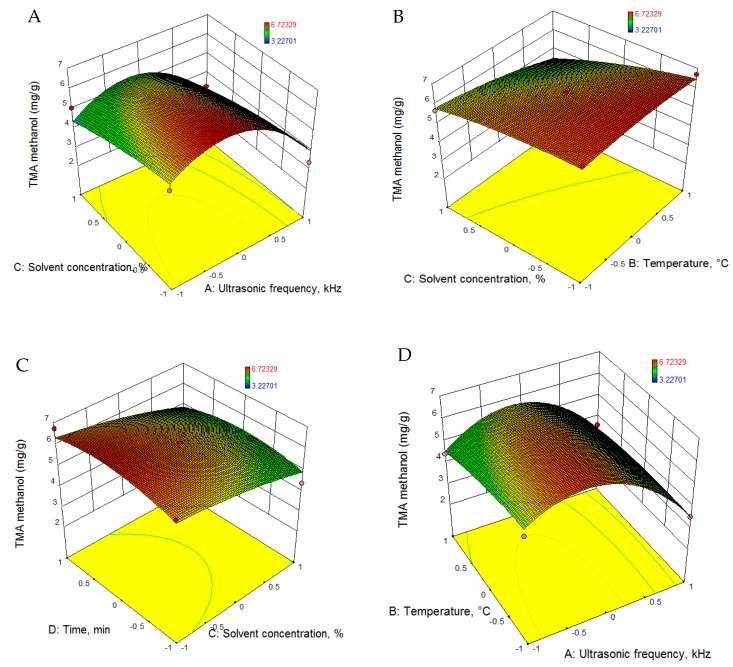

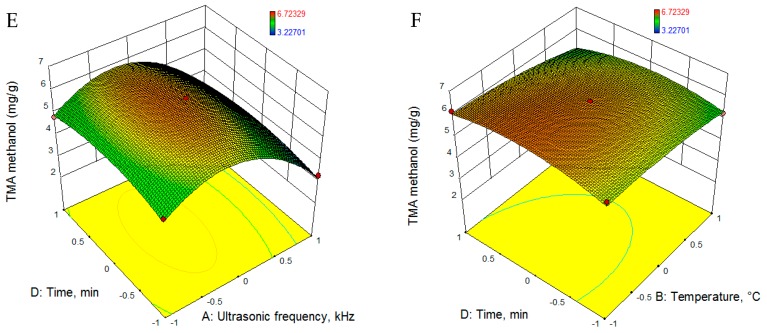
RSM graphs of TMA extraction evolution using methanol as solvent.

Experimental data for TMA was fitted to quadratic equations using the RSM, and the equations are presented in Table 5. Statistical parameters, namely, sum of squares, mean square, F-ratio, R-squared and lack of fit values, were calculated for each model. Based on the statistical parameters presented in Table 6, all the models proposed are significant (*p* < 0.001). The regression analysis revealed that for both extraction solvents (2-propanol and methanol), the process parameters had significant positive linear effects on TMA. Figure 1 and Figure 2 present the 3D evolution of TMA extraction as a function of the parameters employed.

For the purpose of achieving the maximum extraction yield of TMA, ultrasonic frequency, temperature, solvent concentration and extraction time were optimized. Using the RSM methodology for optimization, 2-propanol was found to be the most suitable solvent, and with a concentration of 51% 2-propanol, 27.5 kHz ultrasonic frequency, a temperature of 61.7 °C and 32.6 min extraction time, the predicted TMA yield will be 7.727 mg/g (d = 1.0).

#### 3.2.2. Total Phenolic Content

**Figure 3:**
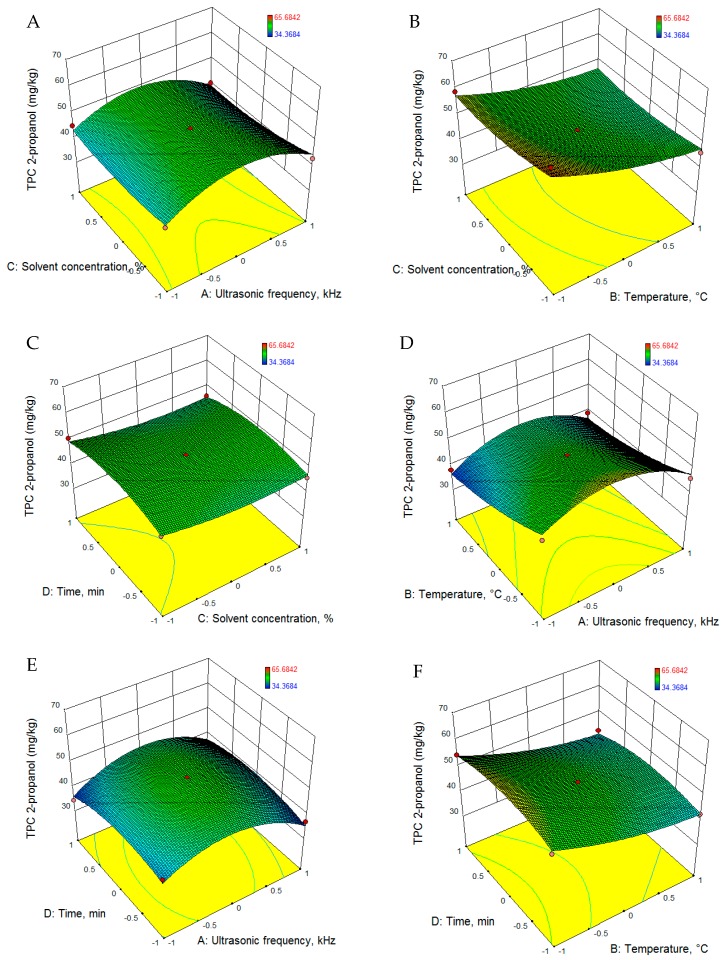
RSM graphs of TPC extraction evolution using 2-propanol as solvent. TPC: total phenolic content.

**Figure 4:**
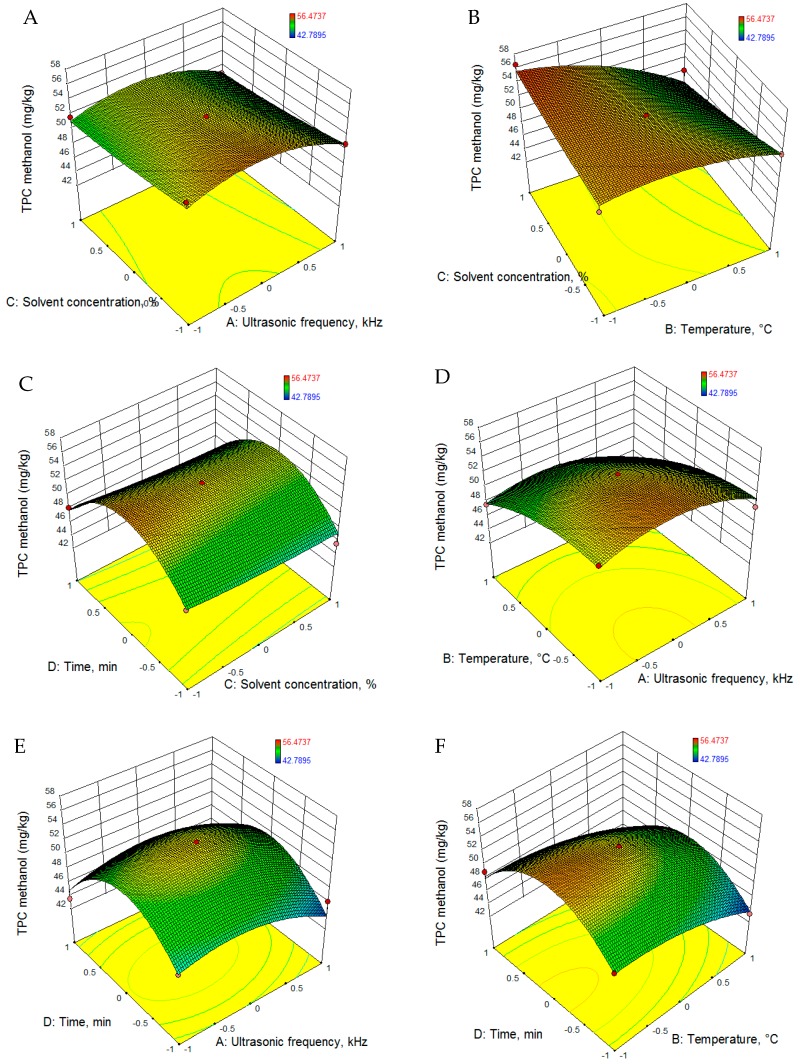
RSM graphs of TPC extraction evolution using methanol as solvent.

The RSM was used for fitting the experimental data with quadratic equations, which are presented in Table 5. According to the values presented in Table 6, all the models proposed are significant (*p* < 0.001). The regression analysis revealed that for both extraction solvents, 2-propanol and methanol, the above-mentioned process parameters had significant positive linear effects on the total phenol content. The 3D graphs illustrating the evolution of TPC extraction as a function of the parameters employed are presented in Figure 3 and Figure 4.

To achieve the maximum extraction yield of TPC, the levels of ultrasonic frequency, temperature, solvent concentration and extraction time were optimized. According to the optimization, in order to achieve the highest yield of TPC (62.487 mg gallic acid equivalent (GAE)/g (d = 1.0)), the following conditions are required: solvent-2 propanol, solvent concentration 50%, temperature−50°C, and extraction time 29.6 min.

### 3.3. Raman Spectra Analysis

Raman spectroscopy is a technique that can be used in the analysis of samples from the wine industry, including grape must, wine and by-products, because it is able to provide an estimation of the sample composition in a matter of only a few seconds. Reported applications of Raman spectroscopy in winemaking include the determination of water, ethanol and sucrose content in white wines, and the rapid measurement of phenolic compounds in red wines [67,68]. In this study, Raman spectroscopy was used to characterize the phenolic compounds contained in two extracts from grape pomace, one with methanol as the extraction solvent, and the other with 2-propanol as the extraction solvent. The levels of other process parameters employed in the extraction of these samples were: solvent concentration of 50%, 37.5 kHz ultrasound frequency, temperature of 60 °C, and 30 min extraction time. The Raman spectroscopy was used to confirm the presence of the phenolics in the extracts.

**Figure 5:**
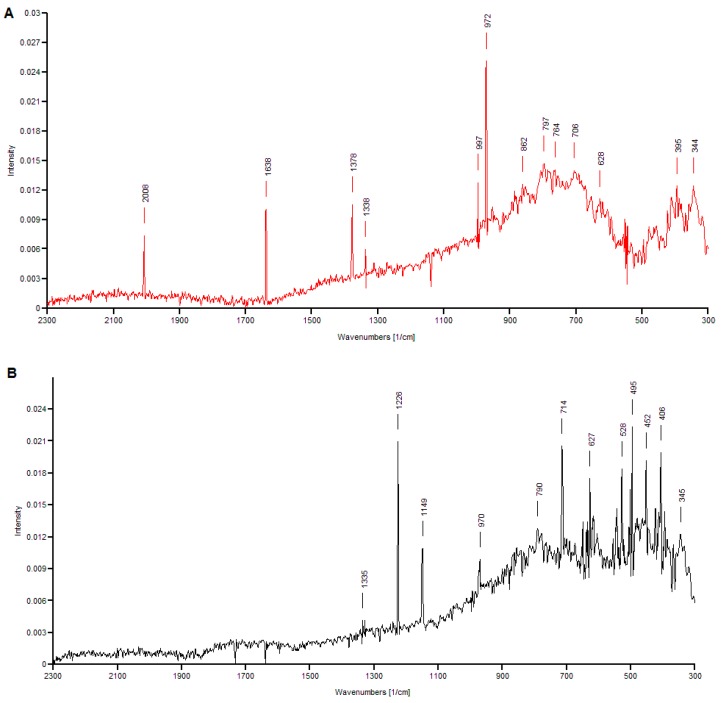
Raman spectra of extracts with 2-propanol (**A**) and methanol (**B**).

The Raman spectra of 2-propanol and methanol extracts from grape pomace are shown in Figure 5 (A-extract with 2-propanol, B-methanolic extract). The spectra of the extract with 2-propanol presented main bands in the wavelength regions 2010–1330 cm^−1^ and 1000–340 cm^−1^ of the electromagnetic spectrum. In the first region, the peak at 2008 cm^−1^ is assigned to the stretching vibration of the hydroxyl (O-H) in A, B, and C ring of anthocyanins. The peak at 1638 cm^−1^ corresponds to the stretching of aromatic C=C, indicating once more the presence of anthocyanins in the extract. The moderate intensity peak at 1378 cm^−1^ could be attributed to the bending of −CH_3_, while the low intensity peak at 1338 cm^−1^ marks the presence of flavonol quercetin, as it is assigned to in-plane O–H bends of the C3−OH group in C ring [69]. Flavonoids are also indicated by the stretch of C−C in the aromatic ring, resulting in the peak at 997 cm^−1^. The high-intensity peak at 972 cm^−1^ is attributed to the stretching of C=C in conjugated C−C=C−C groups, specific to hydroxycinnamic acid, resveratrol and various other phenolic compounds. The bands at 862 cm^−1^ and in the range 800–700 cm^−1^ are assigned to aromatic C−H out-of-plane bending vibrations, especially in ring A. The peak of moderate intensity at 628 cm^−1^ indicates C−C deformation. Lastly, the peak at 395 cm^−1^ corresponds to the bending of C−OH, while the peak at 344 cm^−1^ indicates C−O stretching in all aromatic rings.

The spectra obtained for the methanolic extract from grape pomace (Figure 5B) presented main bands in the wavelength region 1340–340 cm^−1^. The peak of reduced intensity displayed at 1335 cm^−1^ and the high-intensity peak at 1226 cm^−1^ are attributed to C−C stretching in aromatic rings, the latter being specific to the C ring of flavones. The band at 1149 cm^−1^ is assigned to deformation in CH_2_ group [70], while the peak at 970 cm^−1^ is attributed to C−O stretching. The peak at 790 cm^−1^ corresponds to stretching of aromatic C=C. The high-intensity band at 714 cm^−1^ and the moderate intensity bands at 627 cm^−1^ and 528 cm^−1^ are assigned to in-plane deformation in rings A, B and C of flavonoids. The high-intensity peak at 495 cm^−1^ indicates C−C deformation, and the peak at 452 cm^−1^ is particularly assigned to C−C deformation in ring C of flavones. The last two notable peaks of the spectra correspond to vibrations in aromatic rings, as follows: 406 cm^−1^ is assigned to ring deformation, while 345 cm^−1^ indicates ring bending and can also be attributed to in-plane C−O bending. Based on the interpretation of both spectra, it can be concluded that Raman analysis confirms the overall efficiency of methanol in the extraction of total phenolic compounds, and the higher affinity of anthocyanins toward 2-propanol.

## 4. Conclusions

This study focused on the ultrasound-assisted extraction of total monomeric anthocyanis (TMA) and total phenolic content (TPC) from grape pomace resulting as a by-product of processing red grapes from a variety cultivated in the northeastern area of Romania (*Vitis vinifera* Moldova). Two different extraction solvents were used in the extraction, namely, 2-propanol and methanol, and the following process parameters were varied: solvent concentration, ultrasonic frequency, temperature and time. The influence of these experimental parameters on TMA and TPC yields was studied. In TMA extraction, all parameters with the exception of solvent concentration had a positive influence on the extraction yield. In the case of TPC, on the other hand, solvent concentration and temperature had a negative impact on the extraction yield, while the other parameters contributed to the enhancement of the extraction of targeted compounds. For the purpose of reaching the maximum extraction yields of TMA and TPC ultrasonic frequency, temperature, solvent concentration and extraction time were optimized using the RSM methodology. The model predicted that under optimal conditions maximum yields of 7.727 mg/g for TMA and 62.487 mg gallic acid equivalent (GAE)/g for TPC could be achieved. The optimal conditions obtained are based on the design parameters used, but there could be some differences with respect to extraction efficiency if the range of the extraction parameters were changed, taking into account that for some parameters (e.g., temperature, solvent concentration), high concentrations of TPC or TMA could be achieved that were beyond the range studied.

## Figures and Tables

**Table 1:** Levels in full factorial experiments for TMA and TPC from grape pomace.

Factor	−1	0	1
Concentration (%), C	50	70	90
Ultrasonic frequency (kHz), U	12.5	25	37.5
Temperature (°C), T	50	60	70
Time (min), t	15	30	45

**Table 2:** Analysis of variance of TMA and TPC as functions of ultrasonic frequency, solvent type, concentrations, temperatures and times.

Parameter		TMA (mg/g)–Mean Values	TPC (mg/g)–Mean Values
Solvent type	2-Propanol	5.37	42.54
	Methanol	4.58	46.53
ANOVA F-ratio		215.32***	158.92***
Solvent concentration (%)	50	5.40	45.72
	70	5.01	44.65
	90	4.51	43.24
ANOVA F-ratio		92.78***	20.72***
Ultrasonic frequency (kHz)	12.5	4.92	43.52
	25.0	6.03	48.92
	37.5	3.97	41.17
ANOVA F-ratio		493.90***	210.32***
Temperature (°C)	50	5.08	48.76
	60	5.17	45.05
	70	4.68	39.80
ANOVA F-ratio		31.88***	269.92***
Time (min)	15	4.84	43.38
	30	5.24	48.18
	45	4.84	42.05
ANOVA F-ratio		24.73***	138.47***

*** *p*< 0.001.

**Table 3:** Interactions between process parameters and their statistical significance.

Process Parameters Interactions	TMA (mg/g)	TPC (mg/g)
Solvent type × concentration	2.92ns	0.08ns
Solvent type × ultrasonic frequency	27.74***	71.76***
Solvent type × temperature	4.40*	21.54***
Solvent type × time	0.31ns	0.45ns
Concentration × ultrasonic frequency	12.80***	3.37*
Concentration × temperature	0.68ns	6.58***
Concentration × time	0.24ns	0.38ns
Ultrasonic frequency × temperature	5.82***	1.17ns
Ultrasonic frequency × time	0.62ns	1.15ns
Temperature × time	1.12ns	0.47ns

ns *p* > 0.05, * *p* < 0.05, *** *p* < 0.001.

**Table 4:** Experimental and coded values for RSM.

Run	Standard Order	Ultrasonic Frequency, *U*		Temperature, *T*		Solvent Concentration, *C*		Time, *t*	
		Coded	Experimental (kHz)	Coded	Experimental (°C)	Coded	Experimental (%)	Coded	Experimental (min)
1	1	−1	12.5	−1	50	0	70	0	30
2	10	0	25	0	60	0	70	0	30
3	7	0	25	0	60	−1	50	1	45
4	4	1	37.5	1	70	0	70	0	30
5	5	0	25	0	60	−1	50	−1	15
6	2	1	37.5	−1	50	0	70	0	30
7	9	0	25	0	60	0	70	0	30
8	6	0	25	0	60	1	90	−1	15
9	3	−1	12.5	1	70	0	70	0	30
10	8	0	25	0	60	1	90	1	45
11	18	0	25	1	70	1	90	0	30
12	13	−1	12.5	0	60	0	70	1	45
13	15	0	25	−1	50	−1	50	0	30
14	11	−1	12.5	0	60	0	70	−1	15
15	20	0	25	0	60	0	70	0	30
16	19	0	25	0	60	0	70	0	30
17	16	0	25	1	70	−1	50	0	30
18	12	1	37.5	0	60	0	70	−1	15
19	14	1	37.5	0	60	0	70	1	45
20	17	0	25	−1	50	1	90	0	30
21	29	0	25	0	60	0	70	0	30
22	28	0	25	1	70	0	70	1	45
23	21	−1	12.5	0	60	−1	50	0	30
24	27	0	25	−1	50	0	70	1	45
25	23	−1	12.5	0	60	1	90	0	30
26	30	0	25	0	60	0	70	0	30
27	22	1	37.5	0	60	−1	50	0	30
28	26	0	25	1	70	0	70	−1	15
29	25	0	25	−1	50	0	70	−1	15
30	24	1	37.5	0	60	1	90	0	30

**Table 5:** Polynomial equations describing the effect of the independent variables considered on the responses (TMA and TPC), for each extraction solvent (2-propanol and methanol).

Response	Polynomial Equation
Total Monomeric Anthocyanin Content	
TMA_2-p_ (mg/g)	7.104 − 0.282 · *U*− 0.172 · *T* − 0.570 · *C* − 0.026 · *t* − 1.420 · *U*^2^ − 0.506 · *T*^2^ − 0.052 · *C*^2^ − 0.514 · *t*^2^ + 0.373 · *U* · *T* − 0.182 · *U* · *C* + 0.056 · *U* · *t* + 0.047 · *T* · *C* − 0.130 · *T* · *t* + 0.014 · *C* · *t*
TMA_m_ (mg/g)	6.169 − 0.814 · *U* − 0.300 · *T* − 0.632 · *C* + 0.053 · *t* − 1.701 · *U*^2^ − 0.187 · *T*^2^ − 0.179 · *C*^2^ − 0.438 · *t*^2^ + 0.246 · *U* · *T* + 0.084 · *U* · *C* + 0.014 · *U* · *t* − 0.347 · *T* · *C* − 0.129 · *T* · *t* − 0.074 · *C* · *t*
Total Phenolic Content	
TPC_2-p_ (mg/g)	49315.79 − 763.15 · *U* − 6265.81 · *T* − 1541.22 · *C* − 421.12 · *t* − 8689.46 · *U*^2^ + 2227.60 · *T*^2^ + 2069.74 · *C*^2^ − 3465.81 · *t*^2^ + 802.63 · *U* · *T* − 1157.89 · *U* · *C* + 1407.89 · *U* · *t* + 1060.52 · *T* · *C* − 118.64 · *T* · *t* − 184.21 · *C* · *t*
TPC_m_ (mg/g)	53105.26 − 1027.19 · *U* − 2798.24 · *T* − 863.94 · *C* − 570.35 · *t* − 2406.97 · *U*^2^ − 1550.39 · *T*^2^ + 316.57 · *C*^2^ − 5811.05 · *t*^2^ − 381.57 · *U* · *T* + 2.63 · *U* · *C* + 523.68 · *U* · *t* − 1802.63 · *T* · *C* − 105.26 · *T* · *t* + 24.21 · *C* · *t*

**Table 6:** Parameters calculated after implementation of the Box–Behnken design.

Response	Sum of Squares	Mean Square	F-Value	R^2^	Lack of Fit
TMA_2-p_ (mg/g)	21.38	1.53	7.95***	0.8954	2.50
TMA_m_ (mg/g)	34.94	2.50	16.75***	0.9475	1.94
TPC_2-p_ (mg/g)	1254.03	89.57	17.99***	0.8981	64.72
TPC_m_ (mg/g)	399.26	28.52	19.94***	0.9555	18.59

*** *p* < 0.0001.

## References

[1] Yi C., Shi J., Kramer J., Xue S., Jiang Y., Zhang M., Ma Y., Pohorly J. (2009). Fatty acid composition and phenolic antioxidants of winemaking pomace powder. Food Chem..

[2] Kierath T., Wang C. (2013). The Global Wine Industry Slowly Moving from Balance to Shortage.

[3] International Organisation of Vine and Wine (2015). Global Economic Vitiviniculture Data.

[4] Wadhwa M., Bakshi M.P.S., Makkar H.P.S. (2013). Utilization of Fruit and Vegetable Wastes as Livestock Feed and as Substrates for Generation of Other Value-Added Products.

[5] Chouchouli V., Kalogeropoulos N., Konteles S.J., Karvela E., Makris D.P., Karathanos V.T. (2013). Fortification of yoghurts with grape (Vitis vinifera) seed extracts. LWT Food Sci. Technol..

[6] Pinelo M., Arnous A., Meyer A.S. (2006). Upgrading of grape skins: Significance of plant cell-wall structural components and extraction techniques for phenol release. Trends Food Sci. Technol..

[7] Dwyer K., Hosseinian F., Rod M. (2014). The Market Potential of Grape Waste Alternatives. J. Food Res..

[8] Makris D.P., Boskou G., Andrikopoulos N.K. (2007). Polyphenolic content and in vitro antioxidant characteristics of wine industry and other agri-food solid waste extracts. J. Food Compos. Anal..

[9] Jiang Y., Simonsen J., Zhao Y. (2011). Compression-molded biocomposite boards from red and white wine grape pomaces. J. Appl. Polym. Sci..

[10] Maier T., Schieber A., Kammerer D.R., Carle R. (2009). Residues of grape (Vitis vinifera L.) seed oil production as a valuable source of phenolic antioxidants. Food Chem..

[11] Mirzaei-Aghsaghali A., Maheri-Sis N., Mansouri H., Razeghi M.E., Safaei A.R., Aghajanzadeh-Golshani A., Alipoor K. (2011). Estimation of the nutritive value of tomato pomace for ruminant using in vitro gas production technique. Afr. J. Biotech..

[12] Llobera A., Cañellas J. (2007). Dietary fibre content and antioxidant activity of Manto Negro red grape (Vitis vinifera): Pomace and stem. Food Chem..

[13] Tseng A., Zhao Y. (2013). Wine grape pomace as antioxidant dietary fibre for enhancing nutritional value and improving storability of yogurt and salad dressing. Food Chem..

[14] Rondeau P., Gambier F., Jolibert F., Brosse N. (2013). Compositions and chemical variability of grape pomaces from French vineyard. Ind. Crop. Prod..

[15] Zacharof M.-P. (2016). Grape Winery Waste as Feedstock for Bioconversions: Applying the Biorefinery Concept. Waste Biomass Valoriz..

[16] López-Vázquez C., Bollaín M.H., Moser S., Orriols I. (2010). Characterization and differentiation of monovarietal grape pomace distillate from native varieties of Galicia. J. Agric. Food Chem..

[17] Arrieta-Garay Y., Blanco P., López-Vázquez C., Rodríguez-Bencomo J.J., Pérez-Correa J.R., Lopez F., Orriols I. (2014). Effects of distillation system and yeast strain on the aroma profile of Albariño (Vitis vinifera L.) grape pomace spirits. J. Agric. Food Chem..

[18] Devesa-Rey R., Vecino X., Varela-Alende J., Barral M.T., Cruz J., Moldes A. (2011). Valorization of winery waste vs. the costs of not recycling. Waste Manag..

[19] Xia E.Q., Deng G.F., Guo Y.J., Li H.B. (2010). Biological Activities of Polyphenols from Grapes. Int. J. Mol. Sci..

[20] Benmeziane F., Cadot Y., Djamai R., Djermoun L. (2016). Determination of major anthocyanin pigments and flavonols in red grape skin of some table grape varieties (Vitis vinifera sp.) by high-performance liquid chromatography–photodiode array detection (HPLC-DAD). OENO One.

[21] Luan L.Y., Zhang Z.W., Xi Z.M., Huo S.S., Ma L.N. (2013). Brassinosteroids regulate anthocyanin biosynthesis in the ripening of grape berries. S. Afr. J. Enol. Vitic..

[22] Amico V., Chillemi R., Mangiafico S., Spatafora C., Tringali C. (2008). Polyphenol-enriched fractions from Sicilian grape pomace: HPLC–DAD analysis and antioxidant activity. Bioresour. Technol..

[23] Ky I., Lorrain B., Kolbas N., Crozier A., Teissedre P.L. (2014). Wine by-products: Phenolic characterization and antioxidant activity evaluation of grapes and grape pomaces from six different French grape varieties. Molecules.

[24] He F., Mu L., Yan G.L., Liang N.N., Pan Q.H., Wang J., Reeves M.J., Duan C.Q. (2010). Biosynthesis of anthocyanins and their regulation in colored grapes. Molecules.

[25] Ratnasooriya C.C., Rupasinghe H.V. (2012). Extraction of phenolic compounds from grapes and their pomace using β-cyclodextrin. Food Chem..

[26] Mazza G., Francis F.J. (1995). Anthocyanins in grapes and grape products. Crit. Rev. Food Sci. Nutr..

[27] Anđelković M., Radovanović B., Anđelković-Milenković A., Radovanović V., Zarubica A., Stojković N., Nikolić V. (2015). The determination of bioactive ingredients of grape pomace (Vranac variety) for potential use in food and pharmaceutical industries. Adv. Technol..

[28] Fontana A.R., Antoniolli A., Bottini R. (2013). Grape pomace as a sustainable source of bioactive compounds: Extraction, characterization, and biotechnological applications of phenolics. J. Agric. Food Chem..

[29] Sousa E.C., Uchôa-Thomaz A.M.A., Carioca J.O.B., Morais S.M.D., Lima A.D., Martins C.G., Alexandrino C.D., Ferreira P.A.T., Rodrigues A.L.M., Rodrigues S.P. (2014). Chemical composition and bioactive compounds of grape pomace (Vitis vinifera L.), Benitaka variety, grown in the semiarid region of Northeast Brazil. Food Sci. Technol..

[30] El Darra N., Grimi N., Vorobiev E., Louka N., Maroun R. (2013). Extraction of polyphenols from red grape pomace assisted by pulsed ohmic heating. Food Biol. Technol..

[31] Pinelo M., Ruiz-Rodríguez A., Sineiro J., Señoráns F.J., Reglero G., Núñez M.J. (2007). Supercritical fluid and solid–liquid extraction of phenolic antioxidants from grape pomace: A comparative study. Eur. Food Res. Technol..

[32] Casas L., Mantell C., Rodríguez M., De La Ossa E.M., Roldan A., De Ory I., Caro I., Blandino A., Cardoso L.C. (2010). Extraction of resveratrol from the pomace of Palomino fino grapes by supercritical carbon dioxide. J. Food Eng..

[33] Aizpurua-Olaizola O., Ormazabal M., Vallejo A., Olivares M., Navarro P., Etxebarria N., Usobiaga A. (2015). Optimization of supercritical fluid consecutive extractions of fatty acids and polyphenols from Vitis vinifera grape wastes. J. Food Sci..

[34] Liazid A., Guerrero R.F., Cantos E., Palma M., Barroso C.G. (2011). Microwave assisted extraction of anthocyanins from grape skins. Food Chem..

[35] Li Y., Han L., Ma R., Xu X., Zhao C., Wang Z., Chen F., Hu X. (2012). Effect of energy density and citric acid concentration on anthocyanins yield and solution temperature of grape peel in microwave-assisted extraction process. J. Food Eng..

[36] Krishnaswamy K., Orsat V., Gariépy Y., Thangavel K. (2013). Optimization of microwave-assisted extraction of phenolic antioxidants from grape seeds (Vitis vinifera). Food Biol. Technol..

[37] Corrales M., Toepfl S., Butz P., Knorr D., Tauscher B. (2008). Extraction of anthocyanins from grape by-products assisted by ultrasonics, high hydrostatic pressure or pulsed electric fields: A comparison. Innov. Food Sci. Emerg. Technol..

[38] Corrales M., García A.F., Butz P., Tauscher B. (2009). Extraction of anthocyanins from grape skins assisted by high hydrostatic pressure. J. Food Eng..

[39] Tao Y., Zhang Z., Sun D.W. (2014). Kinetic modeling of ultrasound-assisted extraction of phenolic compounds from grape marc: Influence of acoustic energy density and temperature. Ultrason. Sonochem..

[40] González-Centeno M.R., Comas-Serra F., Femenia A., Rosselló C., Simal S. (2015). Effect of power ultrasound application on aqueous extraction of phenolic compounds and antioxidant capacity from grape pomace (Vitis vinifera L.): Experimental kinetics and modeling. Ultrason. Sonochem..

[41] Chemat F., Khan M.K. (2011). Applications of ultrasound in food technology: Processing, preservation and extraction. Ultrason. Sonochem..

[42] Teixeira A., Baenas N., Dominguez-Perles R., Barros A., Rosa E., Moreno D.A., García-Viguera C. (2014). Natural Bioactive Compounds from Winery By-Products as Health Promoters: A Review. Int. J. Mol. Sci..

[43] Rabino I., Mancinelli A.L. (1986). Light, Temperature, and Anthocyanin Production. Plant Physiol..

[44] Singleton V.L., Rossi J.A. (1965). Colorimetry of total phenolics with phosphomolybdic-phosphotungstic acid reagents. Am. J. Enol. Vitic..

[45] Cheok C.Y., Chin N.L., Yusof Y.A., Talib R.A., Law C.L. (2013). Optimization of total monomeric anthocyanin (TMA) and total phenolic content (TPC) extractions from mangosteen (Garciniamangostana Linn.) hull using ultrasonic treatments. Ind. Crop. Prod..

[46] Montgomery D.C. (2005). Design and Analysis of Experiments.

[47] Ortega-Regules A., Romero-Cascales I., Ros Garcia J.M., Bautista-Ortin A.B., López-Roca J.M., Fernández-Fernández J.I., Gómez-Plaza E. (2008). Anthocyanins and tannins in four grape varieties (Vitis vinifera L.). Evolution of their content and extractability. OENO One.

[48] Mori K., Goto-Yamamoto N., Kitayama M., Hashizume K. (2007). Loss of anthocyanins in red-wine grape under high temperature. J. Exp. Bot..

[49] Casazza A.A., Aliakbarian B., Mantegna S., Cravotto G., Perego P. (2010). Extraction of phenolics from Vitis vinifera wastes using non-conventional techniques. J. Food Eng..

[50] Khoddami A., Wilkes M.A., Roberts T.H. (2013). Techniques for analysis of plant phenolic compounds. Molecules.

[51] Bart J.C.J. (2005). Additives in Polymers Industrial Analysis and Applications.

[52] Budiul M., Albulescu M. (2013). Comparative study on content of total phenols and flavonoids in grape pomace extracts. New Front. Chem..

[53] Drosou C., Kyriakopoulou K., Bimpilas A., Tsimogiannis D., Krokida M. (2015). A comparative study on different extraction techniques to recover red grape pomace polyphenols from vinification byproducts. Ind. Crop. Prod..

[54] Cheng V.J., Bekhit A.E.-D.A., McConnell M., Mros S., Zhao J. (2012). Effect of extraction solvent, waste fraction and grape variety on the antimicrobial and antioxidant activities of extracts from wine residue from cool climate. Food Chem..

[55] Benmeziane F., Djamai R., Cadot Y., Seridi R. (2014). Optimization of extraction parameters of phenolic compounds from Algerian fresh table grapes, (Vitis vinifera). Int. Food Res. J..

[56] Vardanega R., Santos D.T., Meireles M.A.A. (2014). Intensification of bioactive compounds extraction from medicinal plants using ultrasonic irradiation. Pharmacogn. Rev..

[57] Carrera C., Ruiz-Rodríguez A., Palma M., Barroso C.G., Lovillo M.P. (2012). Ultrasound assisted extraction of phenolic compounds from grapes. Anal. Chim. Acta.

[58] Ghafoor K., Choi Y.H. (2009). Optimization of Ultrasound Assisted Extraction of Phenolic Compounds and Antioxidants from Grape Peel through Response Surface Methodology. J. Korean Soc. Appl. Biol. Chem..

[59] Buckow R., Kastell A., Terefe N.S., Versteeg C. (2010). Pressure and Temperature Effects on Degradation Kinetics and Storage Stability of Total Anthocyanins in Blueberry Juice. J. Agric. Food Chem..

[60] Danisman G., Arslan E., Toklucu A.K. (2015). Kinetic analysis of anthocyanin degradation and polymeric colour formation in grape juice during heating. Food Chem..

[61] Xu D.P., Zhou Y., Zheng J., Li S., Li A.N., Li H.B. (2015). Optimization of ultrasound-assisted extraction of natural antioxidants from the flower of Jatrophaintegerrima by response surface methodology. Molecules.

[62] Zhu Z., Guan Q., Guo Y., He J., Liu G., Li S., Barba F.J., Jaffrin M.Y. (2016). Green ultrasound-assisted extraction of anthocyanin and phenolic compounds from purple sweet potato using response surface methodology. Int. Agrophys..

[63] Goula A.M., Thymiatis K., Kaderides K. (2016). Valorization of grape pomace: Drying behavior and ultrasound extraction of phenolics. Food Bioprod. Process..

[64] Zhang Z.S., Wang L.J., Li D., Jiao S.S., Chen X.D., Mao Z.H. (2008). Ultrasound-assisted extraction of oil from flaxseed. Sep. Purif. Technol..

[65] González-Centeno M.R., Knoerzer K., Sabarez H., Simal S., Rosselló C., Femenia A. (2014). Effect of acoustic frequency and power density on the aqueous ultrasonic-assisted extraction of grape pomace (Vitis vinifera L.)—A response surface approach. Ultrason. Sonochem..

[66] Ghafoor K., Choi Y.H., Jeon J.Y., Jo I.H. (2009). Optimization of Ultrasound-Assisted Extraction of Phenolic Compounds, Antioxidants, and Anthocyanins from Grape (Vitis vinifera) Seeds. J. Agric. Food Chem..

[67] Meneghini C., Caron S., Poulin A.C.J., Proulx A., Emond F., Paradis P., Pare C., Fougeres A. (2008). Determination of Ethanol Concentration by Raman Spectroscopy in Liquid-Core Microstructured Optical Fiber. IEEE Sens. J..

[68] Gallego Á.L., Guesalaga A.R., Bordeu E., González Á.S. (2011). Rapid Measurement of Phenolics Compounds in Red Wine Using Raman Spectroscopy. IEEE Trans. Instrum. Meas..

[69] Teslova T., Corredor C., Livingstone R., Spataru T., Birke R.L., Lombardi J.R., Cañamares M.V., Leona M. (2007). Raman and surface-enhanced Raman spectra of flavone and several hydroxy derivatives. J. Raman Spectrosc..

[70] Ertani A., Pizzeghello D., Francioso O., Sambo P., Sánchez-Cortés S., Nardi S. (2014). Capsicum chinensis L. growth and nutraceutical properties are enhanced by biostimulants in a long-term period: Chemical and metabolomic approaches. Front. Plant Sci..

